# Deep Learning of Orthographic Representations in Baboons

**DOI:** 10.1371/journal.pone.0084843

**Published:** 2014-01-08

**Authors:** Thomas Hannagan, Johannes C. Ziegler, Stéphane Dufau, Joël Fagot, Jonathan Grainger

**Affiliations:** Laboratoire de Psychologie Cognitive, Aix-Marseille University, & CNRS (Centre National de la Recherche Scientifique), Marseille, France; The University of Plymouth, United Kingdom

## Abstract

What is the origin of our ability to learn orthographic knowledge? We use deep convolutional networks to emulate the primate's ventral visual stream and explore the recent finding that baboons can be trained to discriminate English words from nonwords [1]. The networks were exposed to the exact same sequence of stimuli and reinforcement signals as the baboons in the experiment, and learned to map real visual inputs (pixels) of letter strings onto binary word/nonword responses. We show that the networks' highest levels of representations were indeed sensitive to letter combinations as postulated in our previous research. The model also captured the key empirical findings, such as generalization to novel words, along with some intriguing inter-individual differences. The present work shows the merits of deep learning networks that can simulate the whole processing chain all the way from the visual input to the response while allowing researchers to analyze the complex representations that emerge during the learning process.

## Introduction

Baboons can learn to classify strings of letters as real English words or not [1]. Generalization performance to novel words [1] and the existence of transposed letter effects in baboons [2] clearly suggested that the monkeys had learned some kind of orthographic information. However, there is still considerable debate about the precise nature of the information that allows monkeys to achieve such remarkable performance [3], [4]. Are they using purely visual information, unordered strings of letters, or higher-level abstract letter combinations? To shed light on these mechanisms, we used state-of-the-art neural networks of invariant object identification that were designed to simulate the primate's visual ventral stream. The networks were trained with an incremental supervised procedure to perform a lexical decision task on realistic, pixel-based strings of letters using the exact same stimuli and training regime as the baboons in the experiment. We first show that the networks can account for several empirical effects observed in baboons as well as for intriguing inter-individual differences. We then establish that the networks developed ordered letter representations, therefore demonstrating the efficiency of such representations for a purely visually driven system that must learn to tell words and nonwords apart. This study allows us to move one step further towards the goal of separating the “visual” from the “linguistic” in human reading, while setting a new standard for more realistic computational models of visual word recognition and learning.

Because reading is acquired late in development compared to speech, visual words must map onto pre-existing linguistic knowledge [5]. Not surprisingly, theories of reading have assumed an important role of phonology and semantics in orthographic development [6]. The key finding of the Grainger et al. [1] experiment is that orthography, that is, information about letter identity and letter order, can be acquired in the total absence of prior linguistic knowledge in the primate (because neither sounds nor meanings were associated with the letter strings processed by the baboons). How baboons actually performed the task is not known. Current computational models of orthographic coding in human adults essentially involve either letter-based schemes, whereby letters are being assigned positions in the word in more or less flexible ways, or schemes based on letter combinations, whereby, for instance, the word “LIFE” would be represented as the set of bigrams LI, LF, LE, IF, IE, FE (see [7], [8] for reviews). A consensus has not yet emerged within cognitive psychology as to which scheme captures the data in the most parsimonious way. However cognitive neuroscience has provided direct evidence [9], [10] that letter combinations, so-called n-grams, are processed in the Visual Word Form Area - VWFA hereafter, a brain region that specifically responds to orthographic information in humans [11]. Given the solid and long standing evidence that primates represent visual objects by means of combinations of features [12], [13], it appears plausible that baboons might use letter combinations or ngrams to represent written words.

### Deep learning by convolutional networks

In contrast to our claim that baboons use letter combinations or ngrams to perform word/nonword classifications, several authors have now suggested that baboons in the Grainger et al. experiment used exclusively letter-based schemes. For instance, Frost and Keuleers [4] have argued, without a computational proof, that the task could have been performed simply based on the detection of unordered sets of letters in the stimuli. Others have implemented offline classification systems that can learn to classify the letter strings based on letter positions alone, without invoking any type of ngrams [3]. There is often a host of possible explanations to any given phenomenon, and orthographic coding in baboons is no exception. But none of the explanations described above, when they are actually implemented, brings us any closer to understanding how baboons actually solve the task. This is because they all assume either unlimited computational power, or perfect memory resources, or the availability of all training stimuli at once. In addition, these explanations do not attempt to capture the full range of behavior that has now been documented, but instead, focus on one aspect of the data at best. What is needed is a comprehensive computational account consistent with resource-limited brains, with what we know of the primate's visual system, and with systems being subjected to real visual stimuli in an incremental supervised training procedure. Deep convolutional networks possess many if not all of these properties, and are therefore natural candidates for our purpose.

**Figure 1:**
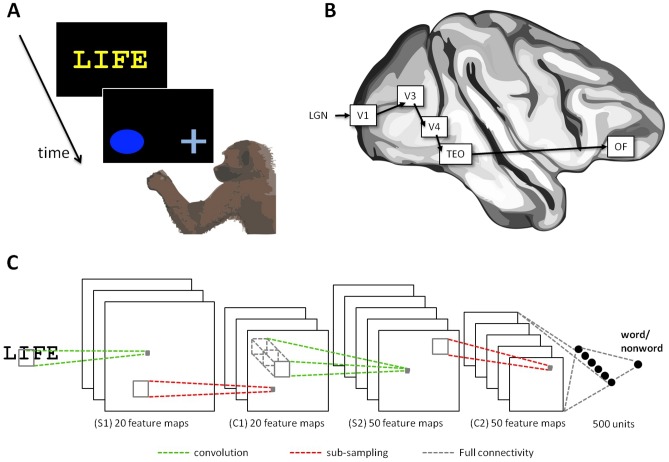
(A) Word-Nonword classification by operant conditioning in baboons [1]. Six baboons were trained to distinguish English words (such as “LIFE”) from nonwords (such as “LEFE”), by touching either the cross or the oval shape presented immediately after the word or nonword. A correct response was followed by a blank screen and a food reward, whereas an incorrect response prompted a green screen to appear for 3s. (B) Main cortical network presumed to be involved in distinguishing between words and nonwords, formed by a succession of increasingly selective regions throughout the ventral stream (LGN-V1-V3-V4-TEO) towards the orbito-frontal cortex (OF). (C) Deep learning convolutional network implementing the local and hierarchical connectivity found in the ventral pathway, through a succession of alternating simple convolution and complex pooling levels (cortical regions not mapped to network levels).

Figure 1 illustrates our general approach. The top left panel (1A) describes the operant-conditioning experiment simulated in this article, in which baboons were shown strings of letters and were rewarded when they classified them accurately as words or nonwords. The top right panel (1B) shows the main pathway presumed to be involved in the baboon's brain when solving this task [14], constituted by a succession of processing stages within the ventral visual stream from V1 to TEO (posterior inferior temporal cortex) and from there to the orbito-frontal cortex. The lower panel (1C) illustrates the convolutional network architecture of the model.

Convolutional networks were initially inspired by the primate's visual system [15], [16]. This is visible in the hierarchical organization of the network Figure 1C, in the restricted connectivity and local sampling of units that captures the increasingly large receptive fields observed from V1 to TEO [17], and in the alternation of convolutional and sampling layers, which mimic the simple and complex cells found in the primary visual cortex. These characteristics have met tremendous success in computer vision and machine learning, and it is fair to say that today convolutional networks display state-of-the-art performance [18], including on tasks such as handwritten character recognition [16], and scene labeling [19]. Closer to our work, the HMAX model, which has been presented in computational neuroscience as the “standard model” of the visual system [20], is very similar in design to convolutional networks.

### The model

The model is a convolutional network with a total of 7 layers, a simplified version of the LeNet5 network [16] built using the Theano deep learning libraries [21]. The first layer receives input images (150×30 pixel units) and the output layer produces a binary decision: Word or Nonword. In between, the S1,C1,S2 and C2 layers implement the succession of convolutional (hereafter S, for simple) and sampling (hereafter C, for complex) stages discussed above.

A convolutional layer consists of a number of feature maps (here, 20 for S1 and 50 for S2). Each unit in each feature map is connected to a restricted patch of units lying directly underneath it in all the maps forming the previous layer (for S1, there is only one map in the preceding layer: the input). Although units in the same feature map are connected to different patches of the previous layer, they all share the same connection weight values, according to the principle that what is learned at one location must hold for all locations (i.e. the principle of “weight sharing”).

Contrary to convolutional layers, sampling layers reduce the dimensionality of the preceding layer. In a sampling layer, each unit in each feature map represents a larger patch from the preceding layer (in our model, patches of dimensions 2×2 units were used), and together all patches form a partition of the sampled layer. The activation of a sampling unit is obtained in the model by a max pooling operator, which outputs the maximally activated unit within the patch considered. After two series of convolution and sampling, the C2 layer is eventually used as input to a standard multilayer perceptron, which has full connectivity from C2 to the hidden layer, and from the hidden layer to the output layer.

Because baboons in [1] were reinforced on each trial during training, an incremental supervised learning procedure was also needed for the networks and we used a stochastic gradient descent algorithm, with the error gradient being evaluated on each trial and a fixed learning rate used at all epochs (annealing of the learning rate may be introduced in future work but was not found necessary to reproduce the baboons learning curves). The error signal in the case of a binary word/nonword classification task like ours amounts to providing always the same reward in case of success (error of 0) and no reward in case of failure (error of 1), which was precisely the training by reinforcement that the baboons had received. In the experiment, new words were introduced one at a time and interleaved with old words and with nonwords. They were shown repeatedly until the baboon succeeded in categorizing a new word more than 80% of the time, at which point the exemplar became a known word and a new word was introduced. Likewise, each network was exposed to exactly the same sequence of word and nonword stimuli as presented to the corresponding baboon. For instance, the “DAN network” (simulating the baboon of the same name) was exposed to 56 689 strings, half being words and half nonwords, with these categories being balanced for positional letter frequencies but differenciated by positional bigram frequency distributions. It should be noted, however, that the six baboons had received plenty of visual experience before the experiment, whereas our networks started out as a blank slate, with random connection weights. We will return to this limit of our model in the Discussion. For this study, assessment of classification accuracy for both baboons and networks was done every 1000 trials during training.

Six networks were instantiated, one for each of the six baboons (DAN, ART, CAU, DOR, VIO, and ARI) trained in the original study [1]. All networks had two free parameters: learning rate and convolution filter size. We launched a brute force exploration of this parameter space on six possible learning rates (from 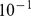 to 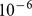) and eight possible filter sizes (from 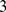 to 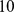 units). Recognition accuracy was collected every 1000 training trials, compared to the corresponding baboon performance, and the parameter values that minimized this distance for each monkey/model pair were selected. Therefore, the models were not fitted to display any generalization, transposition, or letter similarity effect, but only to match the global performance of the baboons during training.

## Results

**Figure 2:**
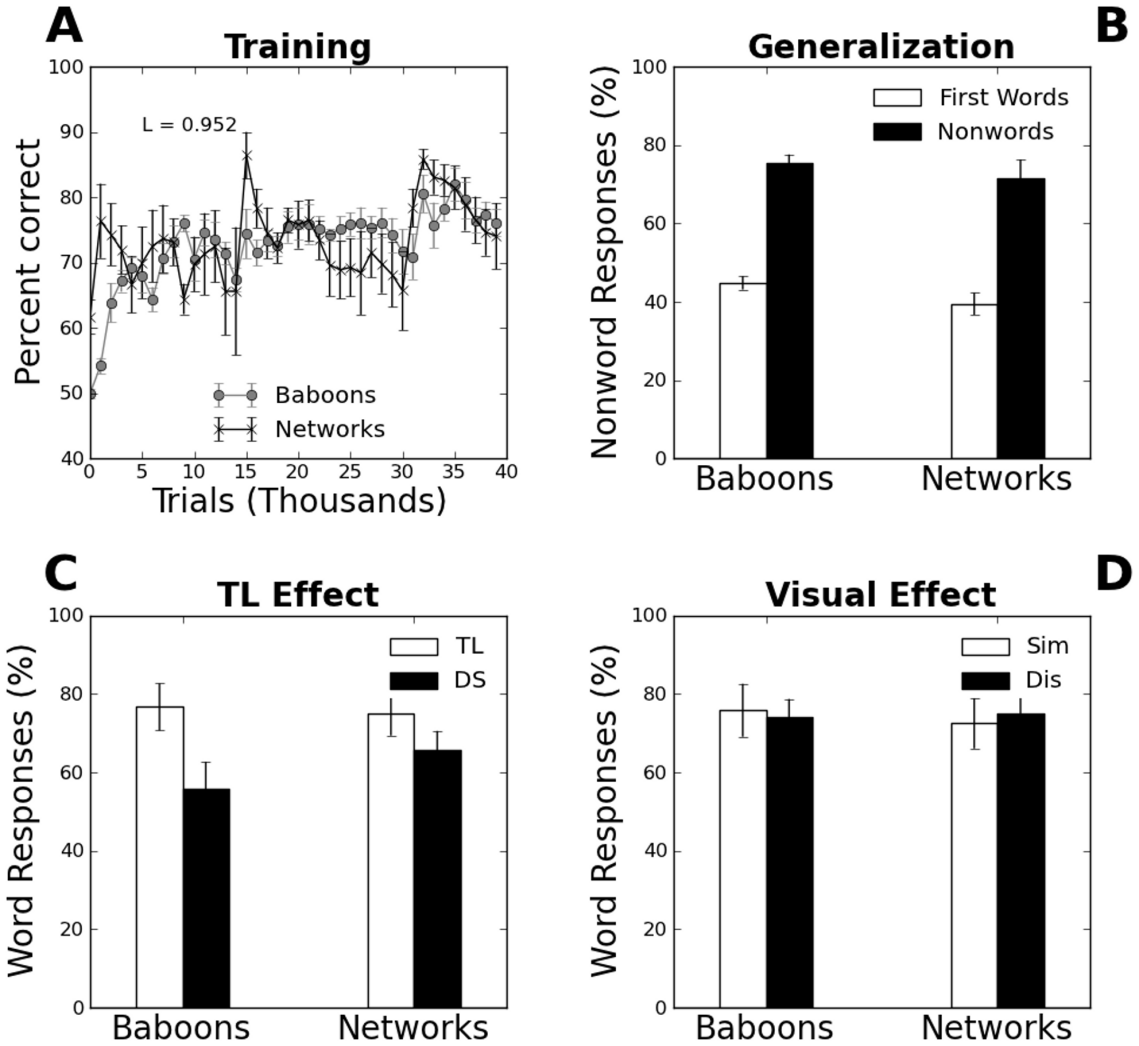
(A) Learning curves in the fitted model, showing the evolution in performance across traning trials in the networks (black crosses) and the baboons (gray dots). Only the first 40 000 trials are shown. (B) Generalization effect: percentage of nonword responses for baboons and networks upon presentation of a new word (white) during training, as opposed to the next nonword (black) in the sequence (experimental data from [1]). (C) Transposition effect: percentage of word responses produced in the networks and in the baboons upon presentation of nonword stimuli that differed from known words by two transposed letters (TL, white bars) or two letter substitutions (DS, black bars) (experimental data from [2]). (D) Visual similarity effect: word responses made on nonword stimuli that were visually similar (Sim, gray bars) or dissimilar (Dis, white bars) to known words (experimental data from [2]).

Figure 2A shows the evolution of performance across the first 40 000 trials, averaged over all fitted networks (black crosses) or baboons (gray filled circles). The main feature of a noisy overall increase in correct responses is captured, and a simple similarity index between model and data based on the absolute difference in performance every 1000 trials approaches the maximal value of 1. By the end of training (not shown in Figure 2A), performance levels were similar though slightly higher for networks (mean accuracy 80.9%) than for baboons (mean accuracy 73.6%).

Figure 2B reports on generalization effects in the networks, that is, the finding that baboons were more likely to classify a real word seen for the first time as a word than a nonword. It can be seen that networks slightly overestimate baboons' performance, but that both are in very close agreement, with much less nonword responses being made upon encountering for the first time a new word (mean baboons 44.8%, mean networks 39.5%) than the next nonword in the sequence (mean baboons 75.6%, mean networks 71.4%). A one-way analysis of variance (ANOVA) on nonword responses for new word stimuli versus nonword stimuli yielded significant differences both for baboons and the model (both ps<.0001). Although a strong similarity in accuracy during training was expected from (indeed the purpose of) our parameter fitting phase, we note that there was no a priori reason why the networks should have displayed quantitatively similar generalization behavior as the baboons, as networks were not fitted to do so.

**Figure 3:**
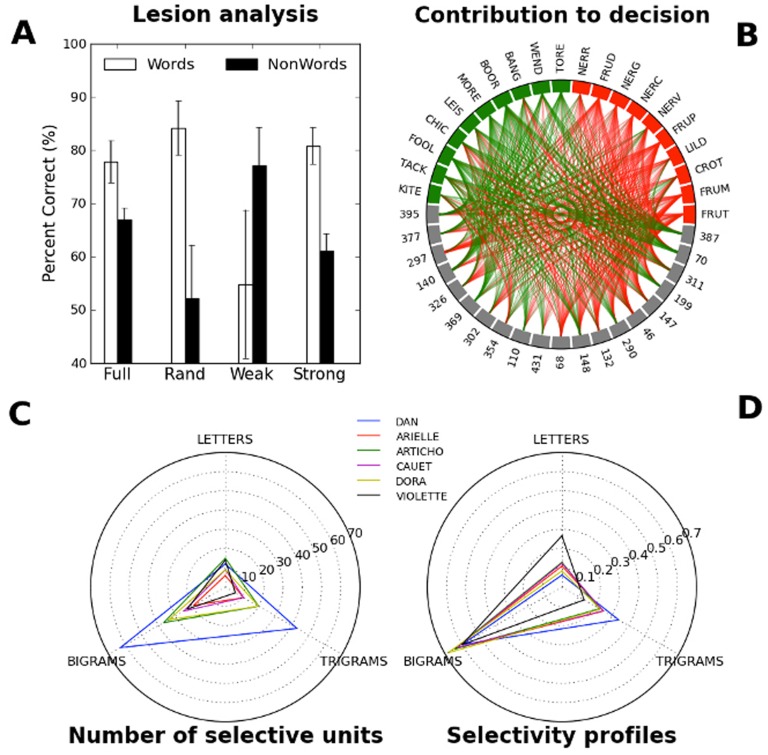
(A) Performance in the normal (Full) and lesioned model, when the 20% intact connections are either chosen at random (Rand), or to be the weakest (Weak) or as the strongest (Strong) connections. (B) Contribution of extremal network units to the lexical decision. Word stimuli occupy the upper left half of the circle, nonwords the upper right half, and the “extremal” units from the last-but-one layer occupy the lower half. A green connection (resp. red) between a unit and a stimulus signifies that this unit contributes a word signal (resp. nonword) to the decision node upon presentation of this stimulus —only 20 units out of 100 are shown. (C) Number of extremal units selective for at least one letter, bigram or trigram, according to a d-prime statistics. A unit was deemed selective for an entity (letter, bigram or trigram) if upon presentation of a word containing or not this entity, the d-prime defined as the difference of z-values for hit rate and false alarm rate reached an arbitrary common threshold. (D) Global proportion of letters, bigrams and trigrams coded for across all extremal units.

We then tested whether the networks displayed letter transposition or letter similarity effects, using the exact same stimuli and conditions as in [2]. Letter transposition effects reflect the finding that transposed-letter (TL) nonwords, such as “caniso”, are more likely to be misclassified as words than controls like “caviro” [22]. 80 nonwords were constructed from four-letter English words for each monkey: 20 were obtained by substituting two letters (condition DS, e.g. LGAE), 20 by transposing two letters (condition TL, e.g. LFIE), 20 by substituting a single letter by another that was visually dissimilar (condition Dis, e.g. LOFE), and 20 by substituting a single letter by a visually similar one (condition Sim, e.g. LJFE). Figure 2C shows the results of our TL simulations, along with the experimental data. The baboons showed a significant TL effect, making more word responses in the TL condition than in the DS but no Visual similarity effect (F(1,5) = 5.2, p = 0.046). The networks also made on average more word responses in the TL than in the DS condition, although this did not reach significance (F(1,5) = 1.56, p = 0.24). Figure 3D reports on the Visual similarity effect: the results show that an equal number of word responses in the Sim and Dis conditions both by baboons (F(1,5) = 0.04, p = 0.84) and by the networks (F(1,5) = 0.09, p = 0.77). Table 1 presents the individual parameters of the networks, their performance on accuracy, generalization, transposition effect, and visual similarity effects, along with the corresponding baboon performance.

**Table 1:** Summary of networks parameters and performance.

	Train. Base		Network Parameters		Network Selectivity			Network Performance				Baboon Performance			
	*Trials*	*Voc.*	*CFilter*	*LRate*	*Let.*	*Big.*	*Tri.*	*Acc.*	*Gen.*	*TL*	*VS*	*Acc.*	*Gen.*	*TL*	*VS*
**DAN**	56 689	308	9	1E-4	12	63	43	91.5	57.09	5	5	79.81	33.7	35	−25
**ARIELLE**	55 407	87	3	1E-5	6	19	11	68.4	9.20	−10	−4.74	71.14	29.7	30	20
**ARTICHO**	61 142	112	3	1E-4	15	37	20	92	40.98	20	20	73.41	26.4	−5	−15
**CAUET**	49 608	121	6	1E-5	9	25	11	70.4	15.18	30	−10	72.43	27.0	0	20
**DORA**	43 041	81	3	1E-4	9	34	20	82.1	35.54	10	−10	73.15	29.5	40	20
**VIOLETTE**	50 985	125	3	1E-4	14	23	6	80.8	33.33	0	15	71.55	38.0	25	−10

Trials: number of trials. Voc: number of words in the training base. Conv. Filter: convolutional filter size. Learn. rate: learning rate. Let, Big and Tri: number of units respectively selective to letters, bigrams and trigrams in the penultimate layer. Acc: accuracy. Gen: generalization effect. TL: transposed letter effect. VS: visual similarity effect.

## Analysis

The fact that deep learning convolutional networks can reproduce several of our key experimental findings, without being fitted to do so, forces us to ask how they achieved this feat. There are a number of properties over and above a deep architecture and supervised learning that might explain the success of these networks in capturing the results —namely the use of weight sharing, feature maps, and interleaved convolutional/pooling stages involving in particular the Max operator. Some of these assumptions are not beyond criticism from the point of view of neuroscience. For instance, doubts have been raised about the classification of cells into simple and complex types [23] —a classification which inspired the convolutional and pooling stages— and even assuming such a classification, there is no evidence for layers of simple and complex cells being interleaved throughout the ventral stream [24], [25]. Other assumptions of convolutional networks appear to be better motivated. For example, Léveillé and Hannagan [26] recently provided a formal analysis of how connection weights suitable for a max operation could arise in networks trained under a Hebbian learning rule with a temporal window. Yet other assumptions, such as weight sharing, are intriguing and would deserve empirical investigation. But regardless of how well-established or plausible they are however, all of these network characteristics fall short of describing the exact learned mechanism or coding scheme whereby the model succeeds in solving the task. Hence a dedicated analysis is called for.

In recent years, the representations formed by deep learning networks have been analyzed in a number of ways. Erhan, Courville, and Bengio [27] proposed, among other strategies, to circumscribe the function of any network unit by launching a systematic exploration of input space in search of the patterns that would maximize the unit's activation. Stoianov and Zorzi [28] were able to determine the selectivity of network units by regressing their activations against some continuous properties of the stimulus, in their case numerosity and cumulative surface area. In what follows we choose a different approach. We contend that a key step towards understanding the model can be taken by partly destroying it. In neuropsychology and computational modeling, lesion studies have provided important insights into cognition [29]–[31], and accordingly we start by asking how the model performs when deprived of 80% of its connections in the penultimate layer — that is, the connections coming from the 500 units that entirely and directly subserve lexical decision.

Figure 3A shows the results of our simulated lesion study. We first tested each trained network's performance on all the words in its training base, and on an equal number of nonwords (condition “Full”). Intact networks reached performance levels that were quantitatively very close to observed accuracies for baboons, both for words and for nonwords (Network mean word accuracy 77.9%, Network mean nonword accuracy 67.0%; Baboons mean word accuracy 76.6%, Baboons mean nonword accuracy 70.0%). We then considered three lesioning conditions, “Rand”, “Weak”, and “Strong”, which had in common the removal of 400 connections and only differed in the 100 being left intact. It can be seen that a lesioned model with all but its strongest connections lesioned (condition Strong) retained essentially the same performance on the training base as an intact model. If anything, performance was slightly improved for words and diminished for nonwords (mean word accuracy 80.8%, mean nonword accuracy 61.2%). When all but 100 randomly selected connections were lesioned (condition Rand), network performance on both words and nonwords was impaired (mean word accuracy 84.1%, mean nonword accuracy 52.2%). Finally when all but the 100 weakest connections were lesioned, performance on words approached chance level whereas performance on nonwords increased (condition Weak, mean word accuracy 54.9%, mean nonword accuracy 77.1%). This analysis suggests that the knowledge used by the model in order to tell whether a stimulus is a word or not is essentially embodied in the 100 strongest connections. Therefore, in order to characterize this knowledge, it is sufficient to restrict the analysis to these 100 “extremal” units that correspond to the strongest connections to the output node. The fact that networks restricted to extremal units can still operate very well is less surprising than it may seem at first blush, being in line with at least one deep learning study, which has found that adequate selection of features in the penultimate stage could actually improve network performance on a task of generic visual object classification [32].

We analyze the connectivity pattern of these extremal units in Figure 3B, where extremal units all lie in the lower half of the circle, word stimuli are in the upper left half, and nonword stimuli in the upper right half. The color of the connection between a given unit and stimulus shows the kind of signal sent by this unit to the decision output upon presentation of the stimulus: a green connection for a positive word signal, and a red connection for a negative word signal. The one fact that immediately stands out in Figure 3B is that word and nonword signals coming from this sample of extremal units are very well matched to word and nonword stimuli. The fact that extremal signals are well matched to lexical status explains why these units are so important in decision making.

Next we assessed the selectivity of extremal units. In this analysis, we exposed each network successively to all the words it had been exposed to and asked whether the observed activity patterns in extremal units showed any regularity with the presence of letters, bigrams, and trigrams in the stimuli at specific positions. We restricted our search to position-specific letters, bigrams, and trigrams, and we adopted two operational definitions for the activity and selectivity of a unit. First, we called a unit *active* if upon presentation of a stimulus, the signal it sent forward to the output layer had a magnitude three standard deviations above the mean across all units and stimuli. Under this count, we found that 15 units were active on average upon presentation of any stimulus, and that a unit was active for on average 22 stimuli. On the other hand, a unit was deemed *selective* for a given entity (letter, bigram, or trigram) if it was mostly active for stimuli that contained this entity and inactive for stimuli that did not contain this entity. We formalized this notion using the d-prime statistic, and granted selectivity to a unit if the difference in Z-scores between hit rate and false alarm rate for the considered entity was superior to an arbitrary and constant threshold (see Methods section for more details).

Figure 3C reports the number of units selective for any letters, bigrams, or trigrams, for each network. It can be seen that every simulated baboon had selective units, with some networks having a majority of their extremal units selective (as in the case of DAN). It is also apparent in this case, because the total sums up to more than 100, that the same same unit can be multiplex, in the sense that it can be selective for several different entities simultaneously. Notably enough, the number of bigram and trigram-selective units in a network did not correlate with the size of the training sets (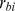 = 0.29; 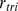 = 0.29), but it strongly correlated with the number of words in them (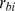 = 0.87; 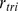 = 0.84), with network accuracy (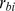 = 0.78; 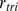 = 0.68), and with generalization performance (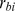 = 0.88; 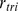 = 0.79). These relations extended to the experimentally observed values in baboons as for accuracy (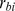 = 0.98; 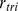 = 0.97), but not for generalization (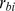 = 0.12; 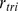 = 0.03). The number of letter-selective units correlated globally in the same way with these variables, only consistently weaker (number of words, 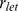 = 0.3; model accuracy, 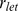 = 0.79; baboon accuracy, 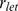 = 0.27; model generalization 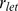 = 0.69; baboon generalization 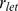 = 0.30). Finally Figure 3D provides an overview of selectivity for each baboon, by displaying the probability that a given selective unit will code for a letter, a bigram, or a trigram. Unlike the absolute number of selective units, it can be seen that the portfolio of entities selected for in each model is quite similar.

### Individual differences: The case of Dan

In our experimental studies, one baboon, Dan, stood out as having acquired the largest vocabulary of all (308 words, more than twice the vocabulary of any other subject), reaching the best accuracy on words (80.0%) and nonwords (79.6%), and showing massive generalization and strong TL effects (33.7 and 35 percentage points, respectively). This is especially puzzling given that Dan was not the baboon who was trained the most (see Table 1). It is striking in Figure 3C and Table 1 that the Dan network is precisely the one that has developed the largest number of bigram and trigram-selective units (resp. 63 and 43 units out of the 100 considered). It appears that the uniqueness of this network can be traced back to the parameter search phase. Although the search yielded a perfectly unremarkable learning rate of 0.0001 for Dan, the size of the convolutional filter was by far the largest, covering 9 units in radius. The straightforward consequence of using a wide convolutional filter is that it allows for a much larger context to be integrated at every convolutional stage, thereby explaining the high number of bigram and trigram selective units that are ultimately available for the Dan network to take decisions upon. Indeed convolutional filter size in the model bore no relationship to the final number of letter-selective units (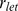 = 0.03), but correlated strongly with the number of bigram (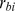 = 0.76) and trigram (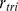 = 0.75) selective units.

## Discussion

This computational study allowed us to propose a general explanation for how baboons can learn to distinguish words from nonwords. Our results suggest that after tens of thousands of training trials the baboons have developed banks of position-specific letter combination detectors. Because words and nonwords in the training base were explicitly designed so as to maximize the difference in bigram frequencies at specific positions, such a strategy explains the baboons' good performance on the training base as well as their ability to generalize to new stimuli, and is well supported by the strong correlation between both of these abilities and the number of bigram selective units. Likewise, our modeling explains why no visual effect was obtained in baboons: if the final decisions were only based on highly selective letter combination detectors in the top layer of the network, then one would expect that the details pertaining to the specific shapes of the letters would have disappeared at this late stage of integration.

Let us now discuss our findings in the light of other recent computational studies of orthographic coding. Two independent analyses on different word recognition networks have now failed to found any support for the emergence of letter combinations, and have instead reported solid evidence for letter-based schemes [33], [34]. Although both networks used artificial letter strings as inputs, the first had a shallow architecture [33], whereas the second used a deep architecture [34]. This suggests that in itself a deep learning design is not sufficient to trigger the emergence of such detectors, and the use of pixel-based visual inputs is unlikely to make a difference in this respect because of one very constraining and critical property shared by these networks: the use of full connectivity between layers. Indeed in both networks, every unit at a given layer was connected to every unit in adjacents layers, effectively forcing every unit to take all the information from the preceding layer into account. In contrast the sparse connectivity used in convolutional networks and in the primate ventral visual stream frees the network from this constraint, and allows each unit to only analyze a limited patch of information. This encourages the specialization of units on different fragments of the pattern from the previous layer, and these fragment detectors in turn get combined to form more complex fragment detectors at the next convolutional step. According to this view, the presence of two critical factors, sparse connectivity and deep architecture, is required in order to produce letter combination detectors.

It coud also be argued that a position-specific letter combination account of orthographic coding in baboons is not entirely compelling in that it does not a priori predict any TL effect, and indeed the networks only showed a non-significant trend towards one, at odds with the observed data. One way to make sense of this conflict is that we may be dealing with a very volatile effect, whose magnitude would depend on minute factors that are not necessarily captured in the model. According to the model, what ultimately determines the presence or absence of a TL effect is not whether the code is position specific or position invariant, but rather, how many more selective units will send a “word” signal to the output layer in the TL condition relative to the DS one. This would depend, first, on the differences in letter, bigram and trigram frequencies between TL and DS stimuli, and second on the exact selectivity profile of the network's units which is a result of the whole training history. It is possible that we made some implausible simplifications during the training phase, for instance by keeping the learning rate and convolution kernel parameters constant throughout training, or by specifying that the perceived reward was always constant. One obvious implausible simplification of this model is that stimuli were always presented in the same position and size on the simplistic simulated retina, whereas actual fixations made by baboons on the letter strings were bound to have varied in locations. Stochastic presentation went beyond the main point of our model, which was essentially to acknowledge the limited resources and hierarchical organization of the primate's visual system. In addition, and due to their built-in weight sharing mechanism for location invariance, deep convolutional networks may not be the best-suited framework for studying the effects of stochastic stimulus presentation. Regardless of this, the distribution of words on the retina may affect orthographic representations, and indeed the finding that orthographic representations in the VWFA are location sensitive has lately been a topic of much interest [35], which some of our computational work has aimed at explaining [36].

We have also trained our networks on a lexical decision task without the benefit of any previous visual expertise, whereas baboons were already experts at recognizing all sorts of visual objects by the time they started experimental training. Introducing previous visual training in this model would be very interesting for a number of reasons, not least because it would allow for the study of mirror invariance, a general characteristic of the expert visual system that has been theorized to be lost during the acquisition of reading [37]. Would the deep learning networks develop mirror-invariance for generic visual stimuli, and would they lose it upon exposure to strings of letters? Regardless of what these answers turn out to be, previous visual training would almost surely impact on the type of features used to perform lexical decision, but we would argue that it is unlikely to threaten our main conclusions here. Indeed, a developmental-like, unsupervised training stage has already been introduced in extant work with deep learning networks on visual categorization, and suggests that such training would result in a dictionary of feature combinations [20]. It is hard to see how the presence of feature combinations at the beginning of training on the lexical task could prevent the emergence of letter combination detectors later on, or could prevent these more developmentally realitistic networks to display any of the effects reported in this study.

We may finally speculate on how the penultimate layer, that was the focus of our analysis, relates to the baboon's brain or to the VWFA. The penultimate layer appears late in the processing stream and its units stand out as the only ones having access to all the information across all feature maps and all locations in the preceeding layer. For that reason, it is perhaps more plausibly related to a region lying between TEO and the orbital frontal cortex. Alternatively, because clear evidence for letter, bigram and quadrigram representations has been found in the VWFA [10], the penultimate layer could be a simplified, structureless homologue of the VWFA itself, whose detailed connectivity is not yet known. In this case, assuming that the same causes produce the same effects in a neural hardware shared across baboons and humans, the model's prediction would be that a region analogue to the VWFA should be observed in baboons that would only be activated for words and legal nonwords but not for false-font letter strings or for illegal, random strings of letters.

## Conclusion

We have used deep learning networks to uncover the nature of the orthographic code which allows baboons to perform word-nonword classifications at a high level of accuracy. To do so, we have applied a range of techniques inspired from neuroscience, especially lesion studies and single cell recordings. The understanding of the model's inner workings gained hereby provides new insights into the nature of the orthographic code in humans. Under the dual pressure of visual processing constraints shared by all primates, and of an incremental training procedure, the model has converged towards a code that uses an assortment of position-specific letter combination detectors in order to categorize letter strings. This is in line with a large but still controversial class of theoretical models of visual word recognition proposed over the last decade [8], [13], [38]–[40], and weighs in favor of similar letter combinations being involved in the human faculty of reading.

## Methods

No new data was collected for this study, this being a computational modeling article that aims at explaining data already published [1], [2]. The previous experiments on baboons had the following characteristics:

- Name of the Institutional Animal Care and Use Committee (IACUC) or ethics committee that approved this study: “comité d'éthique de Marseille pour l'expérimentation animale”.
- Details of animal welfare: Housing conditions; 650 m^2^ outdoor enclosure with various climbing structures connected by tunnels to an indoor enclosure.
- Feeding regimens: Baboons were not food deprived, and received daily food ratio (monkey chow, fruits, vegetables) at 5 pm, water was provided ad libitum.
- Environmental enrichment: Climbing structures, live in social group. Use of the ALDM (“Automated Learning Device for Monkeys”) is a form of behavioral enrichment.
- Steps taken to alleviate suffering: no suffering.
- Methods of sacrifice: none.

### Summary of the experiments

Here, we provide a summary of the two experiments modeled in this paper, and we refer the interested reader to the original studies for more details about the material and procedure [1], [2] Six guinea baboons (Papio papio; 3 females) living in semi-freedom participated in the original word learning experiment of [1]. Baboons were free to access and leave the automated experimental booths whenever they wanted. Self-initiated trials began with the presentation of a stimulus (word or nonword) until the baboon touched the screen, at which point the screen displayed a dark blue cross on the left (correct nonword response) and a light blue oval shape on the right (correct word response). Response was followed by a food reward (dry wheat) when a correct word or nonword response had been given, or followed by a 3 sec delay with a green screen if the response was incorrect. Words and nonwords were four letter strings designed so as to differ in bigram frequencies. Each baboon saw an equal number of words and nonwords, but because of the self-initiated nature of the trials, their total number differed across baboons (DAN: 56 689, ART: 50 985, CAU: 61 142, DOR: 49 608, VIO: 43 041, ARI: 55 407).

Immediately after training, baboons were tested for effects of visual similarity and transposition [2]. The test items were constructed individually for each monkey on the basis of the words that he or she had learned (e.g. LIFE), by substituting two letters (condition DS, e.g. LGAE), transposing two letters (condition TL, e.g. LFIE), by substituting a single letter with another that was visually dissimilar (condition Dis, e.g. LOFE), and by substituting a single letter with a visually similar one (condition Sim, e.g. LJFE). All of these items were reinforced as nonword trials.

### Statistical analysis of the models

For each model, accuracy on word/nonword discrimination and generalization performance were calculated as described in [1], with the only difference being that for practical reasons in our paper these calculations were done every 1000 trials, both for networks and for baboons. Generalization scores were computed as the proportion of nonword responses made for first words minus the proportion of nonword responses made for nonwords. Transposed-letter effects were calculated as the number of word responses produced by TL nonwords minus the number of word responses produced by double substitution nonwords. Visual similarity effects were obtained as the number of word responses made on nonword stimuli that were visually similar to a word, minus the number of word responses made on nonwords that were visually dissimilar to known words. Following Ziegler et al. [2], the statistical analysis performed across models was a standard one-way analysis of variance.

### Lesion analyses

Networks were lesioned after training was complete, by removing 400 connections out of the 500 arriving at the output node. The 100 connections left intact were either chosen to be the strongest (condition “strong”), the weakest (condition “weak”), or random (condition “random”). Strong and weak connections were defined by the absolute value of their weights: the weight of a strong connection had a large absolute value whereas the weight of a weak connection had a small absolute value (i.e. close to zero).

### Sparseness, activity, and selectivity analyses

For each model, we first collected the activation patterns in all 100 extremal units in the last-but-one layer, upon presentation of all the words in the dataset, and of an equal number of nonwords. Because datasets differed across baboons but were exactly identical for a baboon and for its model, the number N of pairs of word/nonword presented varied across models (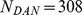; 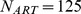; 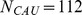; 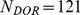; 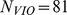; 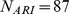). We then computed the mean activation level and standard deviation of all activities across all extremal units and all patterns, and thresholded activation patterns to three standard deviations above the mean - an arbitrary threshold for what counts as an active unit in the model. The resulting Nx100 binary matrix of words-by-units activity served as a base for the computation of sparseness and selectivity in each model (see Figures 3B, 3C and 3D).

Selectivity of a unit to a particular entity (letter, bigram or trigram) was quantified by the d-prime statistic, a signal detection procedure based on the calculation of two quantities: the hit rate (HR), defined as the number of times the unit was active upon presentation of this specific entity divided by the total number of times the entity had been seen, and the false alarm rate (FAR), defined as the number of times the unit was active despite the absence of the entity in the stimulus, divided by this number plus the number of times the unit was not active in the absence of the stimulus (correct rejection). The d-prime was then simply obtained as the difference of z-scores for HR and for FAR, and a unit was deemed selective for an entity if its d-prime was above a common arbitrary value of 2.

All networks were built, trained, and tested using the Theano library [21] running on the Enthought Python Distribution.
